# Telehealth Practice Among Health Centers During the COVID-19 Pandemic — United States, July 11–17, 2020

**DOI:** 10.15585/mmwr.mm6950a4

**Published:** 2020-12-18

**Authors:** Hanna B. Demeke, Leah Zilversmit Pao, Hollie Clark, Lisa Romero, Antonio Neri, Rhea Shah, Kendra B. McDow, Erica Tindall, Naureen J. Iqbal, Kendra Hatfield-Timajchy, Joshua Bolton, Xuan Le, Brionna Hair, Stephanie Campbell, Cuong Bui, Paramjit Sandhu, Isaac Nwaise, Paige A. Armstrong, Michelle A. Rose

**Affiliations:** ^1^CDC COVID-19 Emergency Response; ^2^Oak Ridge Institute for Science and Education, Oak Ridge, Tennessee; ^3^Health Resources and Services Administration, Rockville, Maryland.

Early in the coronavirus disease 2019 (COVID-19) pandemic, in-person ambulatory health care visits declined by 60% across the United States, while telehealth* visits increased, accounting for up to 30% of total care provided in some locations (*1*,*2*). In March 2020, the Centers for Medicare & Medicaid Services (CMS) released updated regulations and guidance changing telehealth provisions during the COVID-19 Public Health Emergency, including the elimination of geographic barriers and enhanced reimbursement for telehealth services^†^ (*3*–*6*). The Health Resources and Services Administration (HRSA) administers a voluntary weekly Health Center COVID-19 Survey^§^ to track health centers’ COVID-19 testing capacity and the impact of COVID-19 on operations, patients, and staff. CDC and HRSA analyzed data from the weekly COVID-19 survey completed by 1,009 HRSA-funded health centers (health centers^¶^) for the week of July 11–17, 2020, to describe telehealth service use in the United States by U.S. Census region,** urbanicity,^††^ staffing capacity, change in visit volume, and personal protective equipment (PPE) supply. Among the 1,009 health center respondents, 963 (95.4%) reported providing telehealth services. Health centers in urban areas were more likely to provide >30% of health care visits virtually (i.e., via telehealth) than were health centers in rural areas. Telehealth is a promising approach to promoting access to care and can facilitate public health mitigation strategies and help prevent transmission of SARS-CoV-2 and other respiratory illnesses, while supporting continuity of care. Although CMS’s change of its telehealth provisions enabled health centers to expand telehealth by aligning guidance and leveraging federal resources, sustaining expanded use of telehealth services might require additional policies and resources.

This analysis used Health Center COVID-19 Survey data for the week of July 11–17, 2020; the response rate was 73.2% (1,009 of 1,379 health centers). Variables included health center location (city, county, state, and urban or rural classification); the percentage of visits through telehealth during the study week; the number of visits during the study week compared with the average number of weekly visits before the pandemic (in 2019); the staffing capacity of health centers during the study week, calculated using data on staff members who were unable to work because of the COVID-19 pandemic, either directly or indirectly (e.g., site or service closure, exposure to COVID-19, COVID-19 symptoms, lack of child care, or lack of PPE); and anticipated adequacy of PPE supplies to serve patients the following week (yes/no). Health centers were categorized into three groups based on the percentage of visits provided virtually using the median value (30%): 0%, ≤30%, and >30%. Log-binomial regression was used to calculate unadjusted prevalence ratios and describe the association between health center characteristics and the percentage of telehealth visits (i.e., those with >30% versus those with ≤30% telehealth visits). P-values <0.05 were considered statistically significant. SAS (version 9.4; SAS Institute) was used to conduct all analyses. This activity was reviewed by CDC and was conducted consistent with applicable federal law and CDC policy.^§§^

Among the 1,009 health centers that completed the July 11–17 COVID-19 survey, 46 (4.6%) reported no telehealth visits, 513 (50.8%) reported >0% but ≤30% telehealth visits, and 450 (44.6%) reported >30% telehealth visits (Table). The proportion of rural health centers varied among Census Regions (Northeast = 30.5%, Midwest = 40.8%, South = 47.0%, West = 40.4%, and U.S. territories and freely associated states^¶¶^ = 65.4%). A higher proportion of health centers in the Northeast (56.1%; prevalence ratio [PR] = 1.81), West (58.9%; PR = 1.90), and U.S. territories and freely associated states (57.7%; PR = 1.86) reported ≥30% telehealth visits than did those in the South (31.1%). Overall, 55.1% of urban health centers reported providing >30% of visits by telehealth compared with 29.9% of rural health centers (PR = 1.84).

**TABLE:** Characteristics of health centers* by percentage of visits provided using telehealth and factors associated with telehealth expansion (N = 1,009) — Health Center COVID-19 Survey, United States, July 11–17, 2020

Characteristic	No. (column %)	No. (%) of health centers			Bivariate association for reporting >30% telehealth visits^†^	
		% of visits by telehealth				
		0%	>0%, ≤30%	>30%	Prevalence ratio (95% CI)	P-value
Total	1,009 (100)	46 (4.6)	513 (50.8)	450 (44.6)		
**Region**						
Northeast	164 (16.3)	4 (2.4)	68 (41.5)	92 (56.1)	1.81 (1.47–2.22)	<0.0001
Midwest	206 (20.4)	10 (4.9)	120 (58.3)	76 (36.9)	1.19 (0.93–1.51)	
South	338 (33.5)	20 (5.9)	213 (63.0)	105 (31.1)	Referent	
West	275 (27.3)	9 (3.3)	104 (37.8)	162 (58.9)	1.90 (1.57–2.29)	
U.S. territories and freely associated states^§^	26 (2.6)	3 (11.5)	8 (30.8)	15 (57.7)	1.86 (1.29–2.68)	
Urban/Rural classification^¶^						
Urban	588 (58.3)	17 (2.9)	247 (42.0)	324 (55.1)	1.84 (1.56–2.17)	<0.0001
Rural	421 (41.7)	29 (6.9)	266 (63.2)	126 (29.9)	Referent	
Staffing capacity**						
Full capacity	368 (36.5)	31 (8.4)	204 (55.4)	133 (36.1)	Referent	<0.0001
95% capacity	412 (40.8)	10 (2.4)	220 (53.4)	182 (44.2)	1.22 (1.03–1.45)	
≤90% capacity	229 (22.7)	5 (2.2)	89 (38.9)	135 (59.0)	1.63 (1.37–1.94)	
Weekly visits before the COVID-19 pandemic^††^						
Decrease	823 (81.6)	43(5.2)	408 (49.6)	372 (45.2)	Referent	0.71
No change	82 (8.1)	2 (2.4)	45 (54.9)	35 (42.7)	0.94 (0.73–1.23)	
Increase	104 (10.3)	1 (1.0)	60 (57.7)	43 (41.3)	0.91 (0.72–1.16)	
Personal protective equipment (PPE) shortage^§§^						
Yes	121 (12.0)	6 (5.0)	69 (57.0)	46 (38.0)	0.84 (0.66–1.06)	0.12
No	888 (88.0)	40 (4.5)	444 (50.0)	404 (45.5)	Referent	

**Abbreviations:** CI = confidence interval; COVID-19 = coronavirus disease 2019.

* Health centers include “Health Resources and Services Administration–funded Federally Qualified Health Centers, which fall under the Consolidated Health Center Program (Section 1905(l)(2)(B) of the Social Security Act).

^†^ Outcome variable was stratified by percent telehealth performed; percent telehealth performed was dichotomized into 0–30% and >30%. The prevalence ratios represent a model predicting >30% telehealth visits.

^§^ US Census regions are defined based on the 2010 Census regions and divisions of the United States (https://www.census.gov/geographies/reference-maps/2010/geo/2010-census-regions-and-divisions-of-the-united-states.html). U.S. territories and freely associated states with health centers that completed the COVID-19 Survey, July 11–17, 2020, include American Samoa, Guam, Marshall Islands, Federated States of Micronesia, and Puerto Rico.

^¶^ Urban/Rural classification is based on the Federal Office of Rural Health Policy criteria. https://www.hrsa.gov/rural-health/about-us/definition/index.html.

** Health centers reported the percentage of staff unable to work during the study week due to COVID-19-related issues (e.g., site/service closure, exposure, family/home obligations, lack of PPE).

^††^ Health centers reported the number of visits during the past week compared with the average number of weekly visits pre-COVID-19.

^§§^ Health centers reported whether they had adequate PPE to serve patients the following week.

Compared with health centers that reported full staffing capacity, the prevalence of reporting >30% telehealth visits was 22% higher among those reporting 5% staff absence (PR = 1.22) and was 63% higher among health centers reporting ≥10% staff absence (PR = 1.63). No association was detected between the percentage of telehealth visits and PPE shortages for the week following the survey (the week ending July 24), nor was an association found between the percentage of telehealth visits and the change in the number of weekly visits from 2019.

## Discussion

The COVID-19 pandemic has resulted in considerable expansion of telehealth services in the United States, which has facilitated care for a range of conditions and improved access for many underserved areas (*7*). Before the COVID-19 pandemic, lack of uniform coverage policies across insurers and states and obstacles to establishing telehealth in health systems (e.g., high startup costs, workflow reconfiguration, clinician buy-in, and patient interest) led to a slow adoption and use of telehealth services in the United States (*8*). Since the pandemic began, telehealth has been used to triage patients and reduce the impact of patient surge on facilities, address decreased access to health care, conserve PPE, and reduce the transmission of SARS-CoV-2 (*9*).

This analysis includes data from HRSA-funded health centers (i.e., Federally Qualified Health Centers). Health centers are community-based and patient-directed organizations that deliver comprehensive, culturally competent, high-quality primary health care services and provide services regardless of patients’ ability to pay, often reaching underserved communities and populations. HRSA’s Health Center Program*** supports nearly 1,400 health centers that provide comprehensive primary health care to approximately 9% of persons across the United States, including one in five rural residents. This report indicates that the majority of health centers used telehealth services during the COVID-19 pandemic. HRSA has awarded funding to support efforts by health centers to mitigate the impacts of the COVID-19 pandemic. In addition to HRSA funding, the CMS provisions to eliminate geographic barriers and enhance reimbursement for telehealth services enabled health centers to expand telehealth services to continue provision of care, enhance response-related services, and reduce the risk for SARS-CoV-2 transmission (*3*–*6*). As the pandemic continues, health care providers and patients might transition back to in-person care; however, a substantial proportion of health care might continue to be provided through telehealth in the future (*2*). Telehealth service expansion is likely to continue to improve access to health care and enhance the health care system’s capacity to respond to future public health emergencies.

As a critical provider of primary care for underserved populations, health centers can play a major role in expanding telehealth and access to care to ensure continuity of care in rural communities; however, the variation in telehealth expansion by region and urbanicity highlights remaining challenges. Approximately one half of health centers in the South are in rural areas, and most of the barriers faced by rural health centers before the pandemic (e.g., the logistics of implementing telehealth, lack of partners or providers, and limited broadband access) will require long-term solutions (*10*). Access to adequate broadband and audiovisual technologies might remain a challenge, specifically for rural health centers and patient populations.

The findings in this report are subject to at least three limitations. First, the data used in this analysis are based on those provided by health centers that reported data to HRSA for one week (July 11–17, 2020) and might not be representative of all health centers in the United States and U.S. territories and freely associated states. Second, these data might be subject to selection bias because completing the Health Center COVID-19 Survey is voluntary, and health centers that completed the survey during the study week might have characteristics (e.g., greater staffing capacity, differences in COVID-19 burden) that are different from those that did not complete the survey. Finally, the percentage of telehealth visits for a single week might not fully capture the expansion of telehealth or consider COVID-19 incidence during that week.

CDC has issued guidance on telehealth including considerations for health care systems, practices, and providers using telehealth services^†††^ applicable both during and after the COVID-19 pandemic. The guidance provides practical approaches to telehealth that can be used to protect health care personnel, patients, and communities.^§§§^ In addition, CDC hosted Clinical Outreach and Communication Activities calls^¶¶¶^ to provide information to clinicians on telehealth benefits and challenges and to share experiences implementing telehealth in health centers.

The potential exists for health centers to improve access to care with removal of barriers, including increasing broadband access and support. Strategies to expand telehealth services through short-term policies and practice changes under the COVID-19 public health emergency have enabled health centers to expand telehealth by aligning guidance and leveraging federal resources (*3*,*4*). Sustaining expanded use of telehealth services in health centers after the pandemic ends might require additional policies and resources.

**Summary**

**What is already known about this topic?**

Limited data are available on expansion of virtual health care visits (telehealth) among U.S. health centers during the COVID-19 pandemic.

**What is added by this report?**

During July 11–17, 2020, 963 (95.4%) of 1,009 Health Resources and Services Administration–funded health centers that responded to a voluntary weekly survey reported providing telehealth services. Health centers in urban areas were more likely to provide >30% of visits virtually than were those in rural areas.

**What are the implications for public health practice?**

Telehealth is a promising approach to promoting and expanding access to care, especially in the South and rural areas; this cost-effective modality can facilitate public health mitigation strategies and prevent transmission of SARS-CoV-2 and other respiratory illnesses, while supporting continuity of care.

## Footnotes

* Telehealth consists of the use of electronic information and telecommunication technologies to support clinical health care, patient and professional health-related education, public health, and health administration https://www.hrsa.gov/rural-health/telehealth.

† Specific to health centers, the Coronavirus Aid, Relief, and Economic Security (CARES) Act allowed health centers to act as distant sites for telehealth applications for the duration of the emergency. On April 17, 2020, CMS issued guidance allowing health centers including Federally Qualified Health Centers (FQHCs) to receive enhanced Medicare reimbursement for telehealth services, per the provision in the CARES Act. Health centers are able to provide and be reimbursed for Medicare services as a distant site provider via telehealth and health center providers can provide these services from any location as long as they are working for the health center and can provide any telehealth service. https://www.hhs.gov/coronavirus/telehealth/index.html.

§
https://bphc.hrsa.gov/emergency-response/coronavirus-health-center-data/ .

¶ Data from HRSA funded health centers (i.e., Federally Qualified Health Centers [FQHCs]) are included for the analysis presented. The term health center is used to include both FQHCs and Health Center Program Look-Alikes. A Health Center Program Look-Alike is a health center that meets all Health Center Program requirements but does not receive Federal award funding. During COVID-19, HRSA provided one-time COVID-19 funding to FQHCs and Health Center Look-Alikes to support COVID-19 testing and response related activities.

** https://www.census.gov/geographies/reference-maps/2010/geo/2010-census-regions-and-divisions-of-the-united-states.html. *Midwest:* Illinois, Indiana, Iowa, Kansas, Michigan, Minnesota, Missouri, Nebraska, North Dakota, Ohio, South Dakota, and Wisconsin; *Northeast:* Connecticut, Maine, Massachusetts, New Hampshire, New Jersey, New York, Pennsylvania, Rhode Island, and Vermont; *South*: Alabama, Arkansas, Delaware, District of Columbia, Florida, Georgia, Kentucky, Louisiana, Maryland, Mississippi, North Carolina, Oklahoma, South Carolina, Tennessee, Texas, Virginia, and West Virginia; *West*: Alaska, Arizona, California, Colorado, Hawaii, Idaho, Montana, Nevada, New Mexico, Oregon, Utah, Washington, and Wyoming; *US territories and freely associated states:* American Samoa, Federated States of Micronesia, Guam, Republic of the Marshall Islands, Puerto Rico, Republic of Palau, and U.S. Virgin Islands.

†† Urban/Rural classification is based on the Federal Office of Rural Health Policy criteria. https://www.hrsa.gov/rural-health/about-us/definition/index.html.

§§ See e.g., 45 C.F.R. part 46, 21 C.F.R. part 56; 42 U.S.C. §241(d); 5 U.S.C. §552a; 44 U.S.C. §3501 et seq.

¶¶ U.S. territories and freely associated states with health centers that completed the COVID-19 survey, July 11–17, 2020: American Samoa, Guam, Republic of the Marshall Islands, Federated States of Micronesia, and Puerto Rico.

***
https://bphc.hrsa.gov/sites/default/files/bphc/about/healthcenterfactsheet.pdf .

†††
https://www.cdc.gov/coronavirus/2019-ncov/hcp/telehealth.html .

§§§
https://www.cdc.gov/coronavirus/2019-ncov/hcp/ways-operate-effectively.html .

¶¶¶
https://emergency.cdc.gov/coca/ .
